# Cutaneous metastasis as the first manifestation of occult malignant breast neoplasia*

**DOI:** 10.1590/abd1806-4841.20164572

**Published:** 2016

**Authors:** Ellem Tatiani de Souza Weimann, Erica Bruder Botero, Cinthia Mendes, Marcel Alex Soares dos Santos, Rafael Fantelli Stelini, Caroline Romanelli T. Zelenika

**Affiliations:** 1 Faculdade de Medicina do ABC (FMABC) – Santo André (SP), Brazil; 2 Private clinic – Araçatuba (SP), Brazil; 3 Private clinic – Tubarão (SC), Brazil; 4 Pontifícia Universidade Católica de Campinas (PUC-Campinas) – Campinas (SP), Brazil; 5 Universidade Estadual de Campinas (UNICAMP) – Campinas (SP), Brazil

**Keywords:** Breast neoplasms, Neoplasm metastasis, Skin

## Abstract

Cutaneous metastases from primary internal malignancies represent 0.7-9% of patients with cancer. We report a 65-year-old female patient referred for evaluation of normochromic papules on the trunk and upper limbs that had been present for three months. A skin biopsy revealed diffuse cutaneous infiltration by small round cell tumors. Immunohistochemistry was positive for AE1/AE3, CK7, estrogen receptor and mammaglobin. The final diagnosis was cutaneous metastasis of occult breast cancer, since the solid primary tumor was not identified. The location of the primary tumor can not be determined in 5-10% of cases. In these cases, 27% are identified before the patient’s death, 57% at autopsy, and the remaining 16% can not be located.

## INTRODUCTION

Cutaneous metastasis is defined as a neoplastic lesion affecting the dermis or the subcutaneous tissue that originates from another primary tumor.^1^ Three basic patterns of metastasis mechanisms are reported: mechanical tumor stasis (anatomical proximity and lymphatic drainage), organ-specific (selective affinity of tumor cells to a specific organ), and nonselective (independent of mechanical and organ-specific factors).^1^

Malignant neoplasms that most commonly metastasize to the skin include breast cancer, colon cancer, melanoma, lung cancer, ovary cancer, sarcomas, and cervical cancer.^1^ In most cases, cutaneous metastasis develops after the diagnosis of the primary internal malignancy and late in the course of the disease. An interval of five years from the initial diagnosis to the skin metastases is common.^2^ 0.7-9% of patients with cancer develop skin metastasis, which is considered a rare dermatological event.^2,3^ However, with the increased incidence of internal cancer, dermatologists may be the first to discover the disease.^2^ A high index of clinical suspicion is essential for the diagnosis of cutaneous metastatic lesions.^3^

## CASE

Who was referred to our institution for evaluation of asymptomatic papules and nodules on the trunk and upper limbs that had been present three months before the consultation. The patient was unable to report the initial morphology or changing pattern of the lesions. She also reported weight loss, which was not measured, and asthenia. Remarkable personal history included anemia treated with ferrous sulfate and a sectorectomy of a benign left breast lump eight years before – which was anatomopathologically confirmed. The patient was G4P3A1 and had been in menopause for 16 years. She denied alcohol abuse, smoking, or remarkable family history.

**Figure 1:**
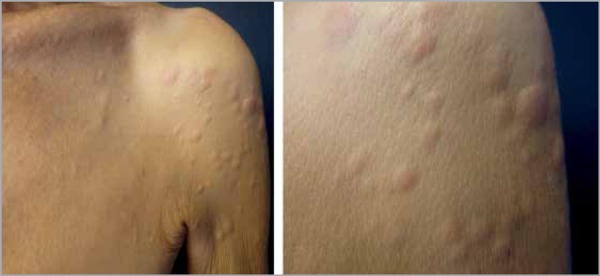
Papules and nodules on the left arm root

**Figure 2:**
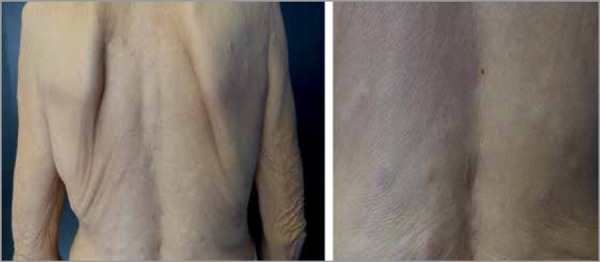
Papules and nodules on the back

**Figure 3:**
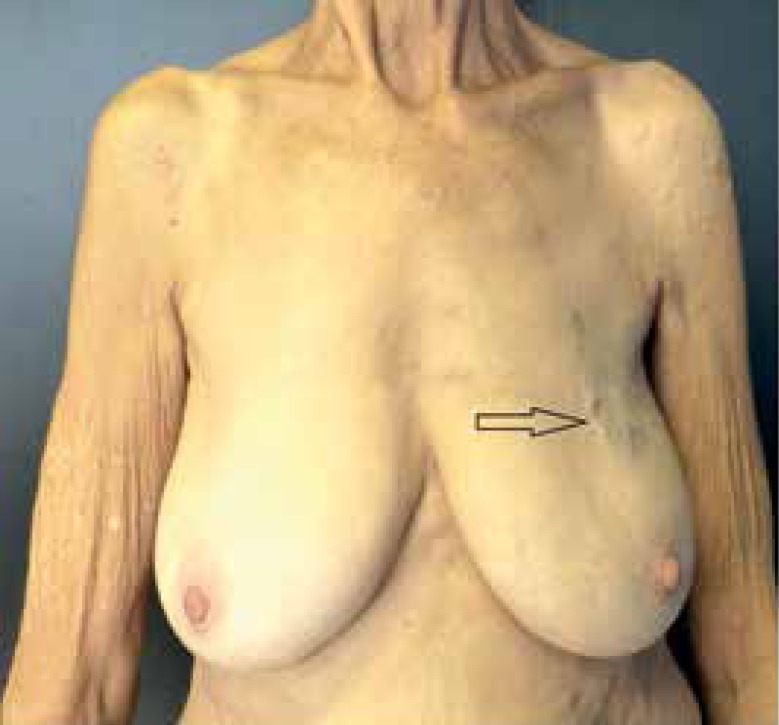
Scar of previous sectorectomy

On physical examination, the patient was emaciated, pale (3+/4+), presented with normochromic rounded papules and nodules, with well-defined regular edges and fibroelastic consistency. The lesions were slightly movable, 0.3-1 cm in diameter, located on the arm root, chest and back. We also observed a linear pearl-colored scar on the left breast (Figures 1-3).

**Figure 4:**
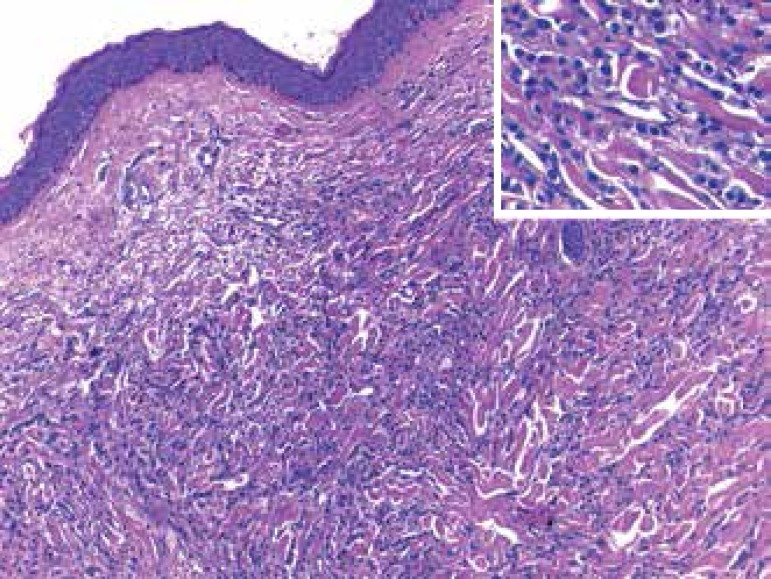
Skin: diffuse neoplastic infiltration in the dermis (hematoxylin and eosin; x200); Inset: higher magnification showing small round cell tumors

Additional tests were performed (Chart 1). Histopathology of two areas of the skin showed a diffuse round cell infiltration in the dermis and part of the hypodermis, sparing the epidermis, divulsing the collagen bundles. The lesions were isolated or linearly arranged in rows, printing an irregular pattern (Figure 4). Immunohistochemical examination showed strong and diffuse positivity for AE1/AE3, favoring the diagnosis of carcinoma, and negative results for S100 protein and leukocyte common antigen (LCA). Melanoma and hemolymphatic cancers were ruled out. Positive multigene panel showed positivity to CK7, mammaglobin, and estrogen receptor, suggesting the breast tissue as the primary site. Other markers are shown in Table 1. The patient was referred to a gynecologist who, after a clinical evaluation, opted for a biopsy of axillary lymph nodes on the left side: four lymph nodes with metastatic carcinoma.

**Chart 1:** Results of additional tests and complementary immunohistochemistry

**Blood count and biochemistry**
•Hgb: 7.2 g%, PCV: 24%, MCV: 116 pg
•ESR: 135mm
•CA 125: 63.4 (=32)
•CEA: 12.1 (=9)
**Imaging exams**
•Thoracic CT: axillary lymph node on the left;
•Endoscopy: erosive esophagitis grade a (LA Classification).
Positive H. pylori
•Normal chest X-ray, mammography, tomography of the abdo-
men/pelvis, and colonoscopy.
**Immunohistochemistry - Other Markers**
•TIF-1, CK20, CDX2, WT1, CD43, p63, C-kit and CD34: negative.

**Table 1:** Histopathological features of melanomas

	Abdomen	Back	Scalp
Histological type	Superficial spreading	Superficial spreading	Superficial spreading
Breslow	0.10 mm	Non-applicable	0.60 mm
Ulceration	Absent	Absent	Absent
Mitotic rate	0/mm^2^	Non-applicable	0/mm^2^
Clark level	II	I	III

Given the clinical findings and laboratory test results, a final diagnosis of cutaneous metastasis of occult breast cancer was made since the primary solid tumor was not detected by any other examination. We reviewed the slides of the sectorectomy performed in 2005 and identified no neoplasias. The patient was then referred to an oncologist for follow-up and chemotherapy. Unfortunately, she died 10 months after the diagnosis.

## DISCUSSION

The frequency of cutaneous metastases has increased due to higher cancer survival rates and better therapeutic alternatives.^4^ The identification of both the skin cancer and the primary tumor can be difficult due to the great variability in clinical course. After a cancer treatment, metastases can be the first sign of relapse, having a significant prognostic value for substantially reducing survival rates.^2^

Metastases are more common in older people. A skin biopsy in patients with cancer should be considered in case of early or sudden onset, delayed healing, bleeding tendency, or vascular appearance of the lesions that do not resolve after treatment.^2^

About 60% of the metastatic cancers are adenocarcinomas. Malignant neoplasms that most metastasize include breast, lung, and gastrointestinal tract cancers. In patients under 50 years of age, metastasis is often linked to malignant melanoma. In pediatric patients, although infrequent, metastasis are usually caused by neuroblastomas and leukemias.^5,6^

The most common sites of metastasis (75%) are the scalp, navel, chest wall, and abdomen, and in 75% of women, they occur on the chest and abdomen. In women, the most common primary malignancy is breast cancer (69%), which tends to *metastasize* later to the anterior thoracic wall.^3,7^ Van den Hurk *et al*. (2011) analyzed metastases patterns in patients diagnosed with primary breast cancer and found that skin metastases often appear later than metastases in other areas.^8^

Although metastasis clinical presentation is variable, it is more frequently characterized as normochromic or brownish firm nodules with sudden onset. Lesions can be painless or associated with pain and sensitivity, with fast initial growth and subsequent stabilization. They may be solitary or multiple, with inflammatory or sclerotic aspects, and may retract the skin.^2,9^ Extensive cutaneous involvement of metastatic breast cancer may mimic cellulitis or a breast-plate of armor (“en cuirasse” pattern).^3^

Differential diagnosis consists of other similar histologically lesions, benign, inflammatory, or malignant, of different prognostics.^10^ Breast cancer immunohistochemistry reveals a cytokeratins pattern of CK7+/CK20-. Estrogens and progesterone receptors are markers that increase the detection sensitivity of breast cancers.^7^

Despite the imaging techniques and immunohistochemistry, the primary tumor location cannot be determined in 5-10% of cases. In general, patients with metastatic carcinoma of unknown primary site have a worse prognosis. In these patients, the primary tumor is only identified in 27% of cases before death; 57%, at autopsy; and for the remaining 16%, primary tumor can not be identified.^8^
